# Supplementary material for: Content-rich biological network constructed by mining PubMed abstracts
Source: BMC Bioinformatics. 2004 Oct 8;5:147. doi: 10.1186/1471-2105-5-147 (PMC528731; doi:10.1186/1471-2105-5-147)
Supplement: Additional File 5 — The original Chilibot query results of the term "long-term potentiation (LTP)" and 22 other terms, limiting the latest references analyzed to the years 1990, 1995, 2000, and 2004. [file 1471-2105-5-147-S5.bz2 › chilibotAdditionalFile5/ltp2000/html/CREB_ATF.html]

 


 **CREB** and **ATF** 
  
Found 374 abstracts in PubMed,  **30 abstracts were retrieved and analyzed**.  


---

 Search Google  |
 PDF files only 
|  EDU domain only 

---

**Interactive relationship** (e.g. stimulation, inhibition, etc)

- Members of the  **CREB**   **ATF**  1 CREM family of transcription factors bind to TRE 1 and are critical for HTLV 1 transcription.  Ref: 11002260 J Med Virol, 2000
- Using the yeast two hybrid system, we identified an interaction between the BRCA1 RING finger and ATF1, a member of the cAMP response element binding protein activating transcription factor  **CREB**   **ATF**  family.  Ref: 10945975 J Biol Chem, 2000
- It is able to bind directly to target promoters to activate transcription but is also able to bridge between upstream binding factors such as  **CREB**   **ATF**  and the basal transcription complex as well as interacting directly with general transcription factors such as TATA binding protein and TFIIB.  Ref: 10906177 J Virol, 2000
- **CREB**  2 also called ATF4, TAXREB67, or C  **ATF**  is an evolutionarily conserved member of the  **CREB**   **ATF**  family of basic leucine zipper transcription factors.  Ref: 10885750 Dev Biol, 2000
- Further, PDGF, PI 3 kinase, and Rac1 each activated the cyclin D 1 promoter at the cyclic adenosine monophosphate response element binding protein  **CREB**  activating transcription factor  **ATF**  2 binding site, as evidenced by expression of a  **CREB**   **ATF**  2 reporter plasmid.  Ref: 11017907 Am J Respir Cell Mol Biol, 2000

**Parallel relationship** (e.g. studied together, co-existance, homology, etc.)

- During syncytium formation increasing protein expression of activating transcription factor  **ATF**  1, cAMP response element binding protein  **CREB**  1, and AP 2alpha was observed on Western blots.  Ref: 11014230 Endocrinology, 2000
- The total level of  **CREB**  1,  **ATF**  2, and  **ATF**  3 was notably reduced.  Ref: 11082295 Exp Cell Res, 2000
- Moreover, phosphorylated  **CREB**  1 and  **ATF**  1 accumulated between 24 and 78 h of trophoblast cultivation.  Ref: 11014230 Endocrinology, 2000
- We further examined expression and activity of  **CREB**   **ATF**  family members to examine their roles in cyclin A inhibition.  Ref: 11082295 Exp Cell Res, 2000
- Phosphorylated  **CREB**  1  **ATF**  1 and the coactivator protein CBP were primarily detected in syncytial nuclei, suggesting the presence of functional, cAMP dependent transcriptional complexes in the differentiated tissue.  Ref: 11014230 Endocrinology, 2000
- We conclude that differentiation dependent transcription of the CGalpha gene in villous trophoblasts is mainly governed by increasing expression of AP 2alpha and PKA dependent phosphorylation of  **CREB**  1 and  **ATF**  1.  Ref: 11014230 Endocrinology, 2000
- This region binds RARs and RXRs nuclear factors,  **CREB**   **ATF**  factors, and also PPARgamma despite the absence of a consensus peroxisome proliferator response element.  Ref: 10921912 J Biol Chem, 2000
- The authors previously reported that one of the cAMP response elements CREs of the human beta3 AR gene, beta3CRE2, interacts with a nuclear factor which is distinct from  **CREB**   **ATF**  family.  Ref: 10940487 Mol Cell Endocrinol, 2000
- This element is recognized by members of  **CREB**   **ATF**  as well as C EBP family of transcription factors.  Ref: 11108139 Mol Cell Biochem, 2000
- Critical to family selective dimerization are intersubunit hydrogen bonds between basic region residue Tyr and leucine zipper residue Glu, which are conserved in all  **CREB**  CREM  **ATF**  1 family members.  Ref: 10952992 J Biol Chem, 2000
- An effector role for methylation in Wp silencing was supported by methylation cassette assays of Wp reporter constructs and by bandshift assays, where the binding of two sets of transcription factors important for Wp activation in B cells, BSAP Pax5 and  **CREB**   **ATF**  proteins, was shown to be blocked by methylation of their binding sites.  Ref: 11044091 J Virol, 2000
- The TRE 1s contain a core cyclic AMP response element CRE motif and can be bound by  **CREB** ,  **ATF**  1,  **ATF**  2, and other members of the  **CREB**   **ATF**  superfamily of transcription factors.  Ref: 10954525 J Virol, 2000
- Here, it is reported that LANA interacts with activating transcription factor  **ATF**  4 cAMP response element binding protein  **CREB**  2, a member of the  **ATF**   **CREB**  family of transcription factors, and represses the transcriptional activation activity of ATF4 CREB2.  Ref: 11038375 J Gen Virol, 2000
- Mildly oxidized LDL also activated MAP and Jun kinases and increased p53 and other transcription factors  **ATF**  2, ELK 1,  **CREB** , AP 1.  Ref: 11023984 FASEB J, 2000
- The binding activity of  **CREB**  1 and  **ATF**  2 to the CRE region of the cyclin A promoter was almost completely abolished due to the treatment.  Ref: 11082295 Exp Cell Res, 2000
- **CREB**  1 and  **ATF**  1 in HepG2 cells.  Ref: 11078737 J Biol Chem, 2001
- We found a significant increase in the protein levels of Elk 1,  **CREB**  and  **ATF**  2 in the cerebellar vermis of schizophrenic subjects.  Ref: 10891039 Arch Gen Psychiatry, 2000
- Treatment of DEX to RAW 264.7 cells induced a dose related inhibition of NF kappaB Rel and AP 1 in chloramphenicol acetyltransferase activity, while neither NF IL6 nor  **CREB**   **ATF**  activation was affected by DEX.  Ref: 10936515 Immunopharmacology, 2000
- In agreement to the in vivo situation, phosphorylated  **CREB**  1  **ATF**  1 were observed in nuclei of the differentiated trophoblast cultures.  Ref: 11014230 Endocrinology, 2000
- We assume that down regulation of cyclin A was mediated by the absence of  **CREB**   **ATF**  activation dimers.  Ref: 11082295 Exp Cell Res, 2000
- The activity of the CGalpha promoter as well as  **CREB**  1  **ATF**  1 phosphorylation increased upon elevation of cAMP levels and overexpression of the catalytic subunit of protein kinase A.  Ref: 11014230 Endocrinology, 2000
- Like all other bZIP proteins, MEQ is capable of dimerizing with itself and with a variety of bZIP partners including c Jun, B Jun, c Fos,  **CREB** ,  **ATF**  1,  **ATF**  2, and SNF.  Ref: 11022789 Virus Genes, 2000
- Since the promoter in IL 1beta gene contains binding motifs for NF kappaB Rel, AP 1, NF IL6, and  **CREB**   **ATF** , which appear to be important in LPS mediated IL 1beta induction, the effects of DEX on the activation of these transcription factors were examined.  Ref: 10936515 Immunopharmacology, 2000
- Inhibiting p38SAPK prevented activation of the transcription factors  **ATF**  2 and  **CREB**  and significantly reduced the LPS induced up regulation of CD80, CD83, and CD86, but did not have any significant effect on the LPS induced changes in macropinocytosis or HLA DR, CD40, and CD1a expression.  Ref: 10910920 Blood, 2000
- Here we report that crc encodes a Drosophila homolog of vertebrate ATF4, a member of the  **CREB**   **ATF**  family of basic leucine zipper bZIP transcription factors.  Ref: 10924469 Genetics, 2000
- Finally, PI 3 kinase and Rac1 induced  **CREB**   **ATF**  2 transactivation were each inhibited by catalase.  Ref: 11017907 Am J Respir Cell Mol Biol, 2000
- ILK activation of the cyclin D1 promoter was abolished by point mutation of a cAMP responsive element binding protein  **ATF**  2 binding site at nucleotide 54 in the cyclin D1 promoter, and by overexpression of either glycogen synthase kinase 3beta or dominant negative mutants of  **CREB**  or  **ATF**  2.  Ref: 10915780 J Biol Chem, 2000
- this occurs mainly through activation of the 19 bp  **CREB**   **ATF**  site of the promoter.  Ref: 11062146 Am J Respir Cell Mol Biol, 2000
- Thapsigargin induced  **CREB**  and  **ATF**  1 phosphorylation and activated CRE dependent transcription in MeWo cells.  Ref: 11108132 Mol Cell Biochem, 2000
- Together, these findings implicate  **CREB**   **ATF**  proteins in essential functions during molting and metamorphosis.  Ref: 10924469 Genetics, 2000
- Analysis of the Epstein Barr virus EBV latent membrane protein 1 LMP 1 gene and promoter in Hodgkin s disease isolates selection against EBV variants with mutations in the LMP 1 promoter  **ATF**  1  **CREB**  1 binding site.  Ref: 11091852 Mol Pathol, 2000
- Subsequent activation of the cAMP PK A signaling pathway results in enhanced synthesis and binding of the transcription factor  **CREB**  1  **ATF**  1 to the cAMP responsive elements within the IE enhancer.  Ref: 10873779 Virology, 2000
- These studies showed that progressive deletion of the 19 bp CMV IE repeats containing cyclic AMP response element binding protein activating transcription factor  **CREB**   **ATF**  sites resulted in progressive loss of activity.  Ref: 11062146 Am J Respir Cell Mol Biol, 2000
